# Efficacy and Safety of Low-Dose Versus Standard-Dose Immunotherapy in Advanced Solid Tumours: A Retrospective Real-World Study From a Resource-Constrained Setting

**DOI:** 10.7759/cureus.108640

**Published:** 2026-05-11

**Authors:** Ajay Gupta, Jinesh Singhvi, Aadithya Lakshmi Narayanan, Shuaib Zaidi, Sudhir Shekhawat

**Affiliations:** 1 Medical Oncology, Indraprastha Apollo Hospitals, New Delhi, IND; 2 Surgical Oncology, Indraprastha Apollo Hospitals, New Delhi, IND; 3 Biostatistics, Indraprastha Apollo Hospitals, New Delhi, IND

**Keywords:** immunotherapy, low dose, nivolumab, pembrolizumab, regular dose

## Abstract

Introduction

Immunotherapy (IO) has revolutionised cancer treatment but is fairly expensive. This has prompted interest in the use of low-dose (LD) IO and is an area of active research. This study retrospectively evaluates the efficacy, safety, and survival outcomes of LD and regular-dose (RD) IO in advanced malignancies.

Methods

A total of 53 patients with advanced (n = 2) or metastatic (n = 51) solid malignancies received IO between November 2021 and January 2024 at a tertiary care centre in Northern India. Patients received either LD (n = 42) or RD (n = 11) IO regimens, alone or in combination with other therapies. Treatment outcomes were assessed using the Immune-modified Response Evaluation Criteria in Solid Tumors (imRECIST) criteria, including progression-free survival (PFS), overall survival (OS), and toxicity profiles.

Results

The overall response rate (ORR) was 52.3% in the LD group and 54.5% in the RD group. Clinical benefit rates (CBR) were 61.9% and 54.5%, respectively. Median PFS was 7.97 months (LD) versus 8.73 months (RD), and median OS was 13.26 months (LD) versus 9.6 months (RD). Log-rank comparison did not reach statistical significance for either PFS or OS. As the RD cohort comprised only 11 cases, the study was not powered for comparative inference, and the numerically similar outcomes between groups should not be interpreted as evidence of equivalence.

Durable complete responses (CRs) exceeding one year were observed in bladder, renal, and triple-negative breast cancers with LD. While favourable responses were also seen in other cancers across both cohorts, Grade ≥3 toxicities occurred in 2 of 42 patients (4.8%) in the LD group and 2 of 11 patients (18.2%) in the RD group and were manageable in both cohorts.

Conclusions

LD IO demonstrated good efficacy and safety across multiple tumour types in the real world, especially for malignancies such as bladder, renal, breast, and certain gastrointestinal and head and neck cancers. These findings support the feasibility of LD IO in resource-constrained settings and provide a preliminary rationale for adequately powered prospective studies to formally evaluate dose optimisation strategies in IO.

## Introduction

Immunotherapy (IO) has emerged as a novel and highly potent cancer treatment strategy in recent times, utilising the body’s own immune system to kill cancer cells. Immune checkpoint inhibitors (ICIs), such as nivolumab, pembrolizumab, and atezolizumab, have demonstrated significant therapeutic efficacy in many cancers. However, the high cost of regular-dose (RD) IO poses many challenges in developing countries.

In India, most patients do not have insurance coverage, and very few are able to bear this out-of-pocket expenditure. A large study from India used nivolumab at a 20 mg dose. The resulting treatment costs were <10% of the cost of nivolumab monotherapy at FDA-approved doses [1]. A large retrospective study from India showed that, out of nearly 15,000 patients eligible for IO, only 3% could afford the drugs [2].

Consequently, alternative dosing strategies, such as low-dose (LD) IO, are being studied. However, there is limited evidence comparing the efficacy and toxicity profiles of LD versus RD therapy regimens.

The primary objective of this study was to describe response rates and survival outcomes in patients receiving LD versus standard-dose (SD) IO. Secondary objectives included evaluation of toxicity and feasibility in a real-world setting. This study is exploratory and hypothesis-generating in nature.

## Materials and methods

Study design

This is a single-institution, retrospective observational study in which we reviewed the data of 53 patients with locally advanced inoperable (n = 2) or metastatic (n = 51) solid tumours who were administered IO between November 1, 2021, and January 31, 2024, at Indraprastha Apollo Hospital, New Delhi, India, a tertiary healthcare facility in Northern India.

Inclusion criteria 

Patients were included in the study if they were aged ≥18 years, had a histologically confirmed diagnosis of advanced or metastatic solid tumour, and had received at least one dose of IO. All patients were treated for indications consistent with the National Comprehensive Cancer Network (NCCN) Clinical Practice Guidelines in Oncology for the respective tumour types [3]. Additionally, only those patients with available baseline clinical data and adequate follow-up records were included in the analysis.

Exclusion criteria 

Patients were excluded from the study if their medical records were incomplete or if they were lost to follow-up before response assessment could be performed.

Data collection 

We reviewed the medical records and obtained data on patient demographics, treatment course, response to therapy, and adverse events.

Ethics

Informed written consent was obtained from patients for the procedures and treatments, and for publication of their clinical information in a journal, with due efforts to conceal their identity. Institutional Ethics Committee approval was also taken for the study (Approval No. IAH-BMR/032/03-26).

Statistics

Given the retrospective design, treatment allocation was not randomised and was influenced by clinical and financial considerations. Treatment outcomes were evaluated by assessing for complete response (CR), partial response (PR), stable disease, or progressive disease (PD) according to the Immune-modified Response Evaluation Criteria in Solid Tumors (imRECIST), which accounts for atypical response patterns seen with IO, including pseudoprogression, by requiring confirmation of progression on subsequent imaging before classification as PD [4]. Imaging assessments were performed every 12-16 weeks using fludeoxyglucose-18 (FDG) positron emission tomography (PET) scans. Imaging was also performed in cases of suspected disease progression. In cases with suspected pseudoprogression, repeat imaging within four to eight weeks was required before confirming PD as per imRECIST criteria. The scans were reviewed only by the treating oncologists and radiologists, and there was no central review. 

The analysis also included overall survival (OS) and progression-free survival (PFS). OS was calculated from the initiation of IO to death from any cause. PFS was defined as the time from initiation of IO to disease progression or death. $\text{Overall Response Rate (ORR)} = \frac{\mathrm{CR} + \mathrm{PR}}{\text{total number of patients}}$ was also assessed, as well as $\text{Clinical Benefit Rate (CBR)} = \frac{\mathrm{CR} + \mathrm{PR} + \mathrm{SD}}{\text{total number of patients}}$.

Missing data were handled using available-case analysis, and no imputation methods were applied, given the retrospective design.

Toxicity assessment was performed at each treatment visit based on clinical evaluation and laboratory parameters and was graded using the Common Terminology Criteria for Adverse Events (CTCAE) scale version 5.0. The common toxicities assessed included dermatological, haematological, cardiorespiratory, gastrointestinal, and endocrine-related toxicities, among others [5].

Performance status was assessed using the Eastern Cooperative Oncology Group (ECOG) scale [6]. The ECOG score ranges from 0 to 5, where 0 indicates full activity without restriction, 1 denotes restriction in strenuous activity but ambulatory status with ability to perform light work, 2 represents ambulatory patients capable of self-care but unable to work and active for more than 50% of waking hours, 3 indicates limited self-care with confinement to bed or chair for more than 50% of waking hours, 4 denotes complete disability with total confinement to bed or chair, and 5 represents death.

Survival analysis was performed using Kaplan-Meier estimates, with the PFS and OS curves plotted. The log-rank test was used to compare survival outcomes between the LD and RD IO groups. All statistical analyses were conducted using IBM SPSS Statistics for Windows, Version 29.0 (Released 2022; IBM Corp., Armonk, NY, USA). A two-sided p-value <0.05 was considered statistically significant. Multivariate analysis was not performed due to the limited sample size and number of events.

## Results

The demography of patients included in the study and the IO therapies employed to treat the tumours are summarised in Table 1. Of the 53 patients included, 16 had recurrent disease and 37 had de novo metastatic or advanced disease. The median age was 61 years (range 25-78), with 33 (62.3%) male and 20 (37.7%) female patients. The cohort included all major tumour types: gastrointestinal (n = 18), head and neck (n = 11), genitourinary (n = 8), gynaecological (n = 8), and lung (n = 8).

**Table 1:** Demographics and treatment characteristics of patients with advanced malignancies receiving immunotherapy (N = 53). ECOG: Eastern Cooperative Oncology Group; PS: performance status; IO: immunotherapy

Parameter	Value
Patient Characteristics	
Total number of patients	53
Median age in years (range)	61 (25-78)
Sex	
Male	33 (62.3%)
Female	20 (37.7%)
Disease Stage	
Stage III	2 (3.8%)
Stage IV	51 (96.2%)
ECOG Performance Status	
PS 0-1 (combined)	8 (15.1%)
PS 2	27 (50.9%)
PS 3-4 (combined)	18 (33.9%)
Tumour Recurrence Status	
Recurrent tumour	16 (30.2%)
Non-recurrent tumour	37 (69.8%)
Treatment	
IO Dose Level	
Regular dose IO	11 (20.8%)
Low dose IO	42 (79.2%)
IO Drug Used	
Nivolumab - total	41 (77.4%)
Nivolumab - low dose	37 (69.8%)
Nivolumab - regular dose	4 (7.5%)
Pembrolizumab - total	7 (13.2%)
Pembrolizumab - low dose	2 (3.8%)
Pembrolizumab - regular dose	5 (9.4%)
Atezolizumab - total	4 (7.5%)
Atezolizumab - low dose	3 (5.7%)
Atezolizumab - regular dose	1 (1.9%)
Ipilimumab + Nivolumab - total	1 (1.9%)
Ipilimumab + Nivolumab - low dose	1 (1.9%)
Ipilimumab + Nivolumab - regular dose	0 (0%)
Line of IO Therapy	
1^st^ line	32 (60.4%)
2^nd^ line	13 (24.5%)
3^rd^ line and beyond	8 (15.1%)
IO Treatment Combination	
IO + chemotherapy	36 (67.9%)
IO + targeted treatment	12 (22.6%)
IO alone	5 (9.4%)

Patients with ECOG Performance Status (PS) of 0-4 were included, of whom 35 had ECOG PS 0-2 and 18 had ECOG PS 3-4 (Table 1). Of these 18 patients with ECOG PS 3-4, 14 received LD IO and four received RD IO. The ECOG 3-4 group had a CBR of 61.1% versus 74.3% in the ECOG 0-2 group (p = 0.35), and the corresponding ORR was 55.6% versus 65.7% (p = 0.55).

Treatment cycles varied between 1 and 20 (median of six cycles), with the majority of patients receiving IO as first-line (n = 32, 60.9%) and second-line (n = 13, 24.5%) therapy. IO was employed alongside chemotherapy (n = 36, 67.9%), with targeted therapy (n = 12, 22.6%), or as monotherapy (n = 5, 9.4%) (Table 1).

The IO agents used included nivolumab, pembrolizumab, ipilimumab, and atezolizumab. Patients were divided into two cohorts: LD (n = 42) and RD (n = 11). LD IO was defined as administration of checkpoint inhibitors at ≤50% of the approved fixed or weight-based dose, or use of extended dosing intervals resulting in reduced cumulative dose intensity. Combination regimens were classified as LD when all components were delivered below standard dosing thresholds. SD/RD was defined as dosing consistent with guideline-recommended regimens.

For nivolumab and pembrolizumab, the doses used were 40 mg and 100 mg, respectively. Patients who received atezolizumab at a lower dose - specifically an 840 mg four-weekly dosing schedule instead of the recommended 1680 mg four-weekly schedule - were also included in the LD group. One patient who received nivolumab 40 mg in combination with ipilimumab 1 mg/kg (50 mg) was likewise included in the LD group, as both agents were administered at low doses. A review of the literature confirmed that this combination had been used in another LD IO study, wherein a lower dose of nivolumab (10 mg bi-weekly) was used alongside 50 mg of ipilimumab [7].

Patients in the RD group received standard dosing of nivolumab 3 mg/kg or 240 mg, pembrolizumab 200 mg, and atezolizumab 840 mg/1200 mg/1680 mg at recommended two-, three-, or four-weekly schedules, as per guidelines. Treatment was continued until disease progression, unacceptable toxicity, or patient unwillingness to continue.

Nivolumab was the most commonly used agent overall (n = 42; LD = 38, RD = 4) and was particularly predominant in the LD group. In the RD group, pembrolizumab was the most frequently used agent (n = 7; LD = 2, RD = 5). Atezolizumab (n = 4; LD = 3, RD = 1) and the nivolumab-ipilimumab combination (n = 1; LD = 1) were the other agents employed.

**Figure 1:**
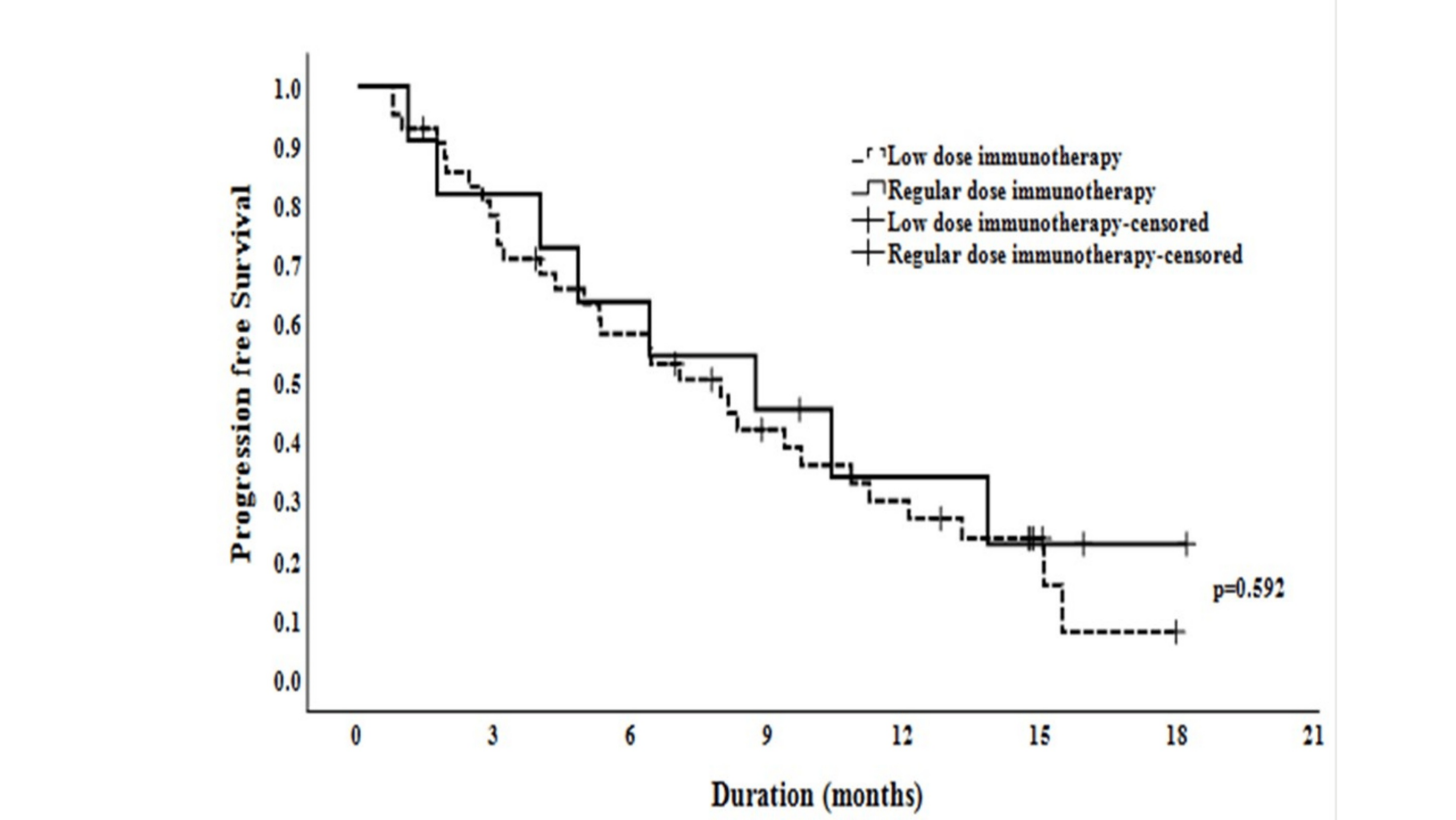
Kaplan-Meier survival curves showing progression-free survival (PFS) in low-dose and regular-dose immunotherapy groups. Kaplan-Meier survival curves for PFS. Comparison performed using the log-rank test.

**Figure 2:**
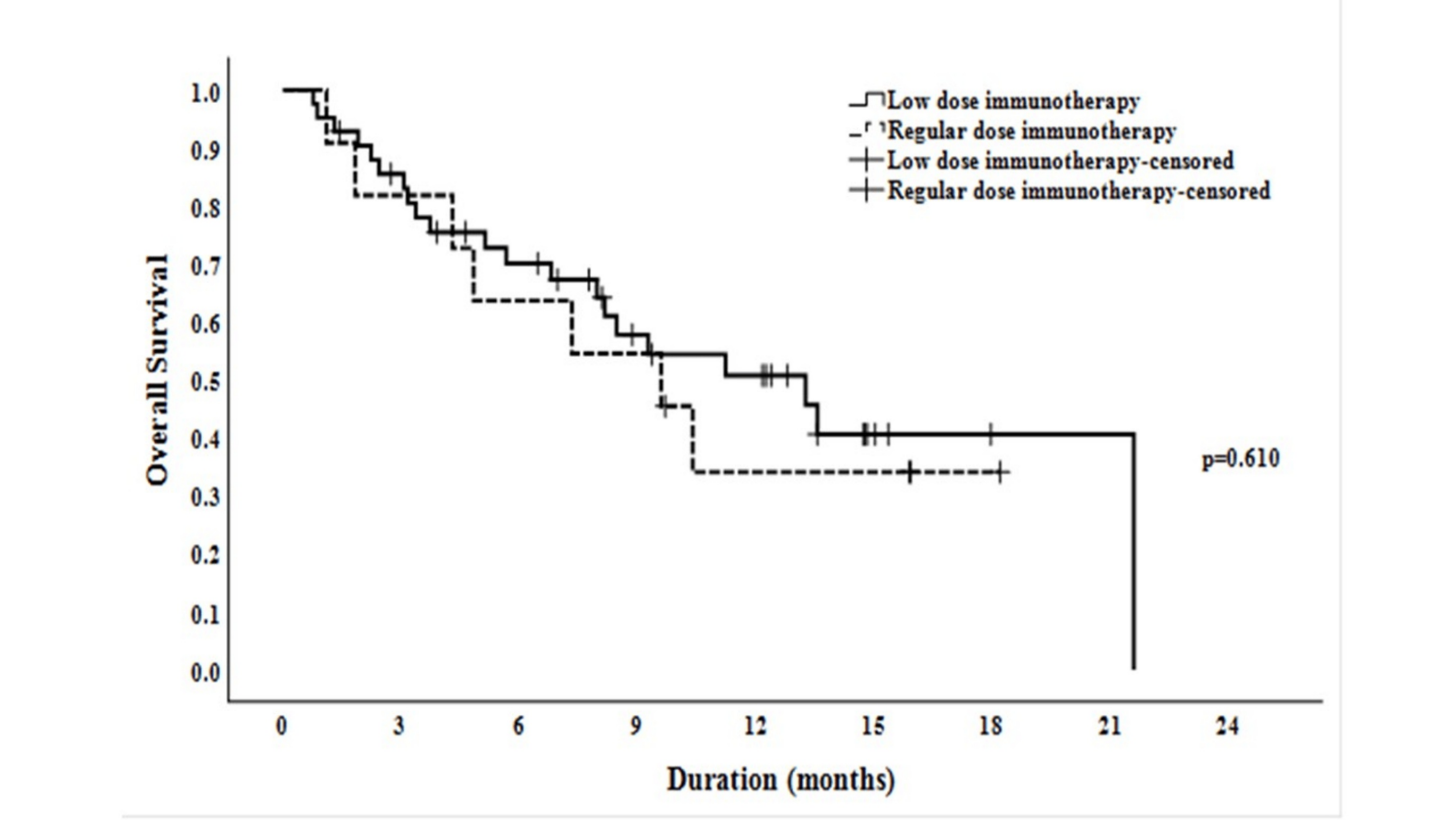
Kaplan-Meier survival curves showing overall survival (OS) in low-dose and regular-dose immunotherapy groups. Kaplan-Meier survival curves for OS. Comparison performed using the log-rank test.

The RD cohort had an ORR of 54.5% (6/11) and a CBR of 54.5% (6/11), with three patients achieving CR (27.2%) and three patients achieving PR (27.2%). Median PFS was 8.73 months (95% CI: 5.7-13 months) and median OS was 9.6 months (95% CI: 6.1-13.9 months) (Figures 1-2). Three patients (27.3%) in the RD cohort achieved PFS >1 year (PFS1) and OS >1 year (OS1) (Tables 2-3).

**Table 2:** Treatment response and survival outcomes by tumour group and dose level - entire cohort (N = 53). Causes of death other than progressive disease are shown in parentheses in the final column. Miscellaneous cancers included anaplastic thyroid cancer (n = 1) and prostate cancer (n = 1). COPD: chronic obstructive pulmonary disease (1 patient, head and neck SCC, regular dose); MI: myocardial infarction (1 patient, bladder transitional cell carcinoma, low dose); PCP: *Pneumocystis jirovecii *pneumonia (1 patient, NSCLC, regular dose); sepsis (1 patient, cervical carcinoma, low dose). CR: complete response; PR: partial response; SD: stable disease; PD: progressive disease; PFS: progression-free survival; OS: overall survival; SCC: squamous cell carcinoma; HNSCC: head and neck squamous cell carcinoma; NSCLC: non-small cell lung cancer; IO: immunotherapy

Tumour group	Total	CR	PR	PFS >1 yr	OS >1 yr	Alive in CR	Alive PR + SD	Alive with PD	Died of disease	Died of other causes
By Tumour Group										
Gastrointestinal	18	1	10	5	6	1	4	4	9	-
Head and neck	10	2	4	2	2	2	2	1	4	1
Genitourinary	7	3	3	3	3	3	1	-	1	1
Gynaecological cancers	8	1	5	1	4	1	1	3	3	1
Miscellaneous cancers	2	-	-	-	-	-	-	-	2	-
Lung	8	-	4	-	1	-	-	2	5	1
Total	53	7	26	11	16	7	8	10	24	4
By Dose Level										
Low dose IO	42	4	23	8	13	4	8	9	19	2
Regular dose IO	11	3	3	3	3	3	0	1	5	2

**Table 3:** Treatment response and survival outcomes by tumour sub-type - regular dose immunotherapy cohort (n = 11). Total number of patients on regular-dose immunotherapy (N = 11). Only tumour subtypes that included patients receiving regular-dose immunotherapy are shown. Deaths attributed to causes other than progressive disease in the regular-dose group (n = 2): 1 patient with HNSCC died of chronic obstructive pulmonary disease (COPD); 1 patient with non-small cell lung cancer (NSCLC) died of *Pneumocystis jirovecii* pneumonia (PCP). CR: complete response; PR: partial response; PFS: progression-free survival; OS: overall survival; IO: immunotherapy; SCC: squamous cell carcinoma; HNSCC: head and neck squamous cell carcinoma; HCC: hepatocellular carcinoma; NSCLC: non-small cell lung cancer

Tumour subtype	Regular dose (n)	CR	PR	PFS >1 yr	OS >1 yr	Alive in CR	Alive with PD	Died of disease	Died of other causes
GI - total	3	1	-	2	2	1	1	1	-
Hepatocellular carcinoma (HCC)	1	-	-	-	-	-	-	1	-
Gastric carcinoma	1	1	-	1	1	1	-	-	-
Oesophageal carcinoma	1	-	-	1	1	-	1	-	-
Head and neck - total	3	2	-	1	1	2	-	-	1
Head and neck SCC (HNSCC)	3	2	-	1	1	2	-	-	-
Lung - total	5	-	3	-	-	-	-	4	1
Non-small cell lung cancer (NSCLC)	5	-	3	-	-	-	-	4	-
Regular dose IO - all tumours	11	3	3	3	3	3	1	5	2

The ORR in the LD group was 52.3% (23/42) and CBR was 61.9% (26/42), with four patients achieving CR (9.5%), 19 patients achieving PR (45.2%), and three patients maintaining stable disease (7.1%). Median PFS was 7.97 months (95% CI: 6.6-10 months) and median OS was 13.27 months (95% CI: 9.9-15.4 months) (Figures 1-2). Survival exceeding one year (OS1) was observed in 13 patients (13/42, 30.9%), and PFS exceeding one year (PFS1) was seen in 11 patients (11/42, 26.1%) (Tables 2-4).

**Table 4:** Treatment response and survival outcomes by tumour sub-type - low dose immunotherapy cohort (n = 42). Cohort low-dose immunotherapy group (N = 42). Deaths attributed to causes other than progressive disease in the low-dose group (n = 2): 1 patient with cervical carcinoma died of sepsis; 1 patient with bladder transitional cell carcinoma died of myocardial infarction (MI). CR: complete response; PR: partial response; SD: stable disease; PD: progressive disease; PFS: progression-free survival; OS: overall survival; IO: immunotherapy; SCC: squamous cell carcinoma; HNSCC: head and neck squamous cell carcinoma; HCC: hepatocellular carcinoma; CRC: colorectal carcinoma; RCC: renal cell carcinoma; NSCLC: non-small cell lung cancer; SCLC: small cell lung cancer

Tumour subtype	Low dose (n)	CR	PR	PFS >1 yr	OS >1 yr	Alive in CR	Alive PR + SD	Alive with PD	Died of disease	Died of other causes
GI - total	15	0	10	3	4	0	4	3	8	-
Hepatocellular carcinoma (HCC)	3	-	1	1	1	-	1	-	2	-
Gastric carcinoma	3	-	3	-	-	-	-	1	2	-
Colorectal carcinoma (CRC)	3	-	2	-	1	-	1	-	2	-
Oesophageal carcinoma	2	-	2	1	1	-	2	-	-	-
Gall bladder carcinoma	1	-	-	-	-	-	-	-	1	-
Pancreatic carcinoma	2	-	1	-	-	-	-	1	1	-
Anal canal carcinoma	1	-	1	1	1	-	-	1	-	-
Head and neck - total	7	0	4	1	1	2	2	1	5	-
Head and neck SCC (HNSCC)	5	-	3	1	1	2	1	1	3	-
Cutaneous SCC	2	-	1	-	-	-	1	-	1	-
GU - total	7	3	3	3	3	3	1	-	2	-
Clear cell renal cell carcinoma (RCC)	3	1	2	1	1	2	1	-	-	-
Bladder transitional cell carcinoma	3	2	1	2	2	2	-	-	-	1
Urothelial carcinoma	1	-	-	-	-	-	-	-	1	-
Gynaecological cancers - total	8	1	5	1	4	1	1	3	3	-
Cervical carcinoma	5	-	4	-	1	-	1	2	2	1
Endometrial carcinoma	2	-	1	-	2	-	-	1	1	-
Breast carcinoma	1	1	-	1	1	1	-	-	-	-
Lung - total	3	-	1	-	1	-	-	2	1	-
Non-small cell lung cancer (NSCLC)	2	-	1	-	1	-	-	2	0	-
Small cell lung cancer (SCLC)	1	-	-	-	-	-	-	-	1	-
Miscellaneous - total	2	-	1	-	1	-	-	2	1	-
Anaplastic thyroid carcinoma	1	-	-	-	-	-	-	-	1	-
Prostate carcinoma	1	-	-	-	-	-	-	-	1	-
Low dose IO - all tumours	42	4	23	13	8	4	8	9	19	2

Biomarker Findings

MSI testing by IHC was performed in 10 patients. One patient with gastric carcinoma had MSI-high status but achieved only a short-lived PR to LD IO. PD-L1 expression was assessed in 12 patients (Ventana SP142 in nine patients and Dako 22C3 in three). PD-L1 <1% was recorded in three patients (one PR and two PD in the LD group); PD-L1 1%-49% in seven patients (one CR, one PR, one SD, and one PD in the LD group, and one CR and two PR in the RD group); and PD-L1 ≥50% in two patients (one CR and one PD, both in the RD group). Given the small number of patients who underwent these tests, no conclusions regarding biomarker-driven response prediction can be drawn.

Response by tumour type

The study covered a variety of solid tumours. The LD cohort included gastrointestinal (n = 15), head and neck (n = 7), genitourinary (n = 7), gynaecological (n = 8), and lung (n = 3) cancers, along with one case each of prostatic carcinoma and anaplastic carcinoma of the thyroid. The RD cohort had fewer cases, consisting predominantly of gastrointestinal (n = 3), head and neck (n = 3), and lung (n = 5) cancers.

Gastrointestinal Tumours (n = 18: 15 LD, 3 RD)

The study covered a variety of solid tumours. The LD cohort included gastrointestinal (n = 15), head and neck (n = 7), genitourinary (n = 7), gynaecological (n = 8), and lung (n = 3) cancers, along with one case each of prostatic carcinoma and anaplastic carcinoma of the thyroid. The RD cohort had fewer cases, consisting predominantly of gastrointestinal (n = 3), head and neck (n = 3), and lung (n = 5) cancers.

Gastrointestinal tumours constituted the largest tumour group. In the RD cohort, one CR was observed in a patient with gastric carcinoma (currently alive and in CR), and one prolonged PR was recorded in a patient with oesophageal carcinoma (currently alive with stable disease). In the LD cohort, the best responses were PRs, observed in three gastric carcinomas, two oesophageal carcinomas, two colorectal cancers and one HCC. PFS1 was achieved in five patients (three LD and two RD) and OS1 in six patients (four LD and two RD). Seven patients died of the disease (five LD and two RD); one gastric carcinoma patient in the LD cohort experienced hyperprogression despite treatment. Of particular note, one HCC patient treated with atezolizumab combined with lenvatinib achieved a prolonged PR with a survival of 27 months at the time of writing (Tables 2-4).

Head and Neck Cancers (n = 10: Seven LD and Three RD)

Favourable responses were seen predominantly in head and neck squamous cell carcinoma (HNSCC) (n = 8), with two CRs in the RD cohort and three PRs in the LD cohort. One PR was also observed in one of the two cutaneous squamous cell carcinoma cases in the LD cohort. PFS1 and OS1 were each achieved by two patients (one in the LD cohort and one in the RD cohort). Two patients with HNSCC in the RD cohort are alive and in CR; one LD patient is alive in PR, one is alive with progressive disease, and one cutaneous SCC patient in the LD group is alive in PR. Five LD patients died of disease progression, and one RD patient died of complications from chronic obstructive pulmonary disease (COPD) (Tables 2-4). 

Genitourinary Tumours (n = 7: All LD)

All genitourinary patients were in the LD cohort. Among the different genitourinary malignancies, bladder transitional cell carcinoma (n = 3) demonstrated the strongest response, with two CRs and one PR; both CR patients are alive and in CR at the last follow-up. Renal cell carcinoma (RCC) (n = 3) yielded one CR and two PRs; the CR patient and both PR patients are alive. PFS1 and OS1 were achieved in three patients (two with bladder carcinoma and one with RCC). These durable responses with 40 mg nivolumab in bladder and renal carcinoma are consistent with published LD nivolumab experience in RCC. Urothelial carcinoma of the renal pelvis (n = 1) derived no benefit, and the patient died of the disease within six months (Tables 2-4).

Gynaecological Cancers (n = 8: All LD)

One CR was observed in a patient with triple-negative breast carcinoma, with PFS exceeding 12 months; this patient remains alive in CR at the last follow-up. Five PRs were recorded: four in cervical carcinoma and one in endometrial carcinoma. PFS1 was achieved in one patient (breast carcinoma), and OS1 in four patients (one breast, one cervical, and two endometrial carcinomas). Three patients are alive with disease progression (two with cervical carcinoma and one with endometrial carcinoma). Three patients died of the disease, and one patient with cervical carcinoma died of sepsis (Tables 2-4).

Lung Cancers (n = 8: Three LDs and Five RDs)

No CRs were observed in either cohort. PRs were recorded in one LD patient and three RD patients, all with non-small cell lung cancer (NSCLC). The single small-cell lung cancer (SCLC) patient in the LD cohort derived no benefit and died of disease within six months. PFS1 was not achieved in any patient, while OS exceeded one year in one patient in the LD group. Two LD patients remain alive despite disease progression. Five patients died of the disease (two LD and three RD), and one patient in the RD group died of *Pneumocystis jirovecii *pneumonia (PCP) following radiotherapy (Tables 2-4).

Miscellaneous

The single case of anaplastic thyroid carcinoma in the LD cohort derived no benefit and died of the disease within five months. One case of prostatic adenocarcinoma also received LD nivolumab but died within two months, having previously exhausted four lines of therapy and having a PD-L1 expression of >10%.

Log-rank comparison of PFS and OS between cohorts did not reach statistical significance (Figures 1-2). As noted, this reflects the study's limited statistical power and cannot be interpreted as evidence of equivalence between the two dosing strategies.

Toxicity

In the LD cohort, toxicities observed included one instance each of Grade 3 anaemia with Grade 1 thrombocytopenia, Grade 1 hypothyroidism, Grade 2 pruritus, Grade 1 hyperthyroidism, Grade 2 hypocortisolism, and Grade 4 neutropenia.

In the RD cohort, toxicities included one case each of Grade 2 generalised pruritus, Grade 3 severe hypothyroidism, and Grade 2 onycholysis followed by Grade 3 severe pneumonitis on rechallenge, the latter two occurring in the same patient.

## Discussion

This study provides real-world evidence on the use of LD IO in advanced solid tumours in a resource-constrained setting. Our findings suggest that reduced-dose regimens may retain clinically meaningful activity in selected patients, with acceptable toxicity profiles. However, the study has a small sample size, particularly in the RD group (n = 11), and was neither designed nor powered to establish equivalence or non-inferiority between the two dosing strategies.

Among the LD cohort, genitourinary cancers had the highest number of CRs, with two transitional cell carcinomas of the bladder and one RCC achieving CR.

In our study, all three patients with RCC received LD nivolumab with cabozantinib and responded well, with one achieving CR and two achieving PR. In pivotal studies of RCC, the ORR with nivolumab plus cabozantinib was 55.7% (95% CI, 50.1 to 61.2), compared to 27.1% (95% CI, 22.4 to 32.3) with sunitinib (p < 0.001), with CRs occurring in 8.0% of patients in the nivolumab-plus-cabozantinib group [8]. Importantly, in both malignant melanoma and RCC, similar ORRs have been reported across nivolumab doses ranging from 0.1 to 10 mg/kg. In a phase 1 trial, patients with melanoma received nivolumab at 0.1, 0.3, and 1 mg/kg, yielding ORRs of 35%, 28%, and 31%, respectively [9]. Notably, patients who did not respond at lower doses also failed to respond at higher doses [9]. A similar pattern was observed in RCC, where nivolumab at doses of 0.3, 2, or 10 mg/kg produced comparable ORRs of 20%, 22%, and 20%, respectively [10]. A retrospective study from Singapore further corroborated these findings, demonstrating that LD nivolumab (100 mg/140 mg) did not compromise efficacy in renal cancers [11].

All three of our patients with bladder transitional cell carcinoma responded to LD nivolumab and chemotherapy, with two achieving CR and one achieving PR, suggesting meaningful activity of the LD IO strategy in this tumour type. In a major study in bladder cancer, the ORR was 57.6% with nivolumab combination therapy versus 43.1% with gemcitabine-cisplatin alone, with CRs in 21.7% and 11.8% of patients, respectively [12].

We observed encouraging and durable CRs in triple-negative breast cancer, RCC, and urinary bladder carcinoma with LD IO, with PFS exceeding one year at last follow-up in each of these cases. Additionally, one patient with gastric cancer and two patients with HNSCC achieved CR in the RD cohort.

In the KEYNOTE-048 study, patients with relapsed HNSCC demonstrated a 36% ORR with the combination of RD pembrolizumab and chemotherapy [13]. A randomised phase III study involving 151 advanced HNSCC patients used LD nivolumab at 20 mg/day in combination with oral metronomic therapy, with a marked improvement in one-year OS from 16.3% to 43.4% and a response rate of 59.2% in the LD IO arm [1]. Our findings were comparable, with an ORR of 62.5% in HNSCC and 60% in the LD subgroup.

In NSCLC, PR was observed in one of two patients in the LD group and in three of five patients in the RD group. The small sample size precludes meaningful comparison, though the results do suggest the efficacy of LD IO. A retrospective study of 47 Korean patients with NSCLC compared LD nivolumab (20 mg/100 mg Q3W) with SD nivolumab (3 mg/kg Q2W) and found similar ORR (16.7% vs 13.8%), PFS (3 vs 1 months), and OS (12.5 vs 8.4 months) [14]. Similar response rates across the dose range of 1 to 10 mg/kg of nivolumab have also been reported in advanced NSCLC [15]. Pooled data from the KEYNOTE-001, -002, and -006 studies showed no difference in response between pembrolizumab doses ranging from 1 to 10 mg/kg every three weeks [16]. LD pembrolizumab has been evaluated in NSCLC across two dedicated studies - one involving 64 patients comparing 2 mg/kg or 100 mg Q3W against 200 mg Q3W [17], and another randomising 114 patients to either 100 mg or 200 mg doses - with both demonstrating comparable PFS and OS [18]. A retrospective study of 150 patients receiving pembrolizumab for NSCLC further showed similar outcomes between extended-interval (>3 weeks + 3 days) and standard-interval groups, suggesting that extended dosing intervals may also represent a viable therapeutic strategy [19].

Among the five cervical cancer patients treated with LD IO, four achieved PR, and one had an OS exceeding one year, suggesting a potential role for LD IO in this disease. In the KEYNOTE-826 trial, pembrolizumab in combination with chemotherapy significantly improved response rates, with an ORR of 65.9%-68% in the pembrolizumab group compared to 50% in the placebo group, with greater benefit in PD-L1-positive tumours [20]. Of the two endometrial cancer patients in our cohort, one on LD IO achieved a PR, and the other had stable disease, with both achieving an OS of more than one year. In the NRG-GY018 trial (pMMR cohort) in advanced endometrial cancer, the ORR in the pembrolizumab plus chemotherapy arm was 55%-60% compared with 45%-50% with chemotherapy alone, with a corresponding PFS of 13.1 versus 8.7 months [21].

Notable responses with LD IO were also seen in GI malignancies, including oesophageal cancer, hepatocellular carcinoma, and anal carcinoma, with durable PRs and stable disease. Favourable responses were additionally observed in gastric, colorectal, and cervical carcinomas.

It has been proposed that IO dosing recommendations should be guided by clinical safety and efficacy data alongside dose-response and exposure-response (D-R/E-R) analyses, rather than fixed dosing alone. With the exception of ipilimumab, no clear dose-efficacy relationship has been established. The dose-response and exposure-response curves demonstrate a clear plateau, suggesting that dose escalation beyond a threshold may not yield incremental benefit and that lower doses may suffice [15,22].

Peripheral PD-1 receptor saturation has been observed with nivolumab at 0.3 mg/kg [23]. For pembrolizumab, 80% receptor occupancy was achieved at 0.2 mg/kg, and 90% at 0.5 mg/kg, with full saturation at 1 mg/kg. Furthermore, pembrolizumab clearance decreases by approximately 20%-30% at steady state compared to clearance after the first dose [24].

For atezolizumab, Genentech initially identified 6 μg/mL as the target plasma concentration, achievable with a 4 mg/kg three-weekly dose in 90% of patients - considerably lower than the standard 1200 mg dose. The labelled dose produces trough levels of approximately 100 μg/mL, nearly 16 times the target concentration. Clinical activity has been documented across doses ranging from 1 to 20 mg/kg every three weeks [25,26]. In silico simulation has further suggested that an 840 mg Q6W intravenous dose maintains therapeutic efficacy in 99% of patients compared to the recommended 840 mg Q2W regimen [27]. Taken together, the pharmacokinetic and exposure-response data support an efficacy plateau beyond certain dose thresholds for PD-1/PD-L1 inhibitors, though it must be noted that peripheral receptor occupancy may not directly reflect drug activity within the tumour microenvironment.

Very high ORRs (the majority of responses being CR) have been achieved with LD pembrolizumab (100 mg every three weeks) and nivolumab (40 mg every two weeks) in relapsed/refractory Hodgkin lymphoma, and re-treatment with the LD regimen has also proved effective [28]. While LD efficacy has been demonstrated in Hodgkin lymphoma, extrapolation to solid tumours should be done cautiously due to distinct tumour biology.

A further study investigated LD nivolumab at 10 mg every two weeks in 18 patients with advanced solid tumours. The ORR was 22% (two CR and two PR), with a CBR of 50%. CRs were observed even in patients negative for PD-L1, tumour-infiltrating lymphocytes (TILs), and high MSI status, suggesting efficacy independent of conventional biomarkers - and representing the lowest reported dose of nivolumab demonstrating clinical benefit [29]. Collectively, these findings support the potential role of LD IO across a broad range of solid tumours.

In the CheckMate 142 trial, nivolumab combined with LD ipilimumab at 1 mg/kg every six weeks was evaluated in MSI-high colorectal cancer, with two-year PFS and OS rates of 53% and 71%, respectively, confirming the long-term efficacy of LD ipilimumab [30].

This study has several limitations. Its retrospective, single-centre design and small sample size - particularly in the RD cohort - limit statistical power and generalisability. The heterogeneity of tumour types and concurrent therapies introduces confounding variables, though this is consistent with the realities of routine clinical practice. Response assessment was not centrally reviewed. Biomarker data, including PD-L1 expression and MSI status, were incomplete and could not be uniformly applied across all patients. The study is therefore hypothesis-generating, and prospective, adequately powered trials are needed to determine whether LD IO is truly equivalent to standard dosing.

The strengths of this study lie in its real-world design, the inclusion of patients with poorer performance status typically excluded from clinical trials, and the representation of diverse tumour types reflective of day-to-day oncology practice in a resource-limited setting.

## Conclusions

This study provides real-world insights into the use of LD and RD IO in advanced solid tumours within a resource-constrained setting. LD IO demonstrated encouraging clinical activity and an acceptable safety profile in this real-world cohort. However, given the exploratory nature and methodological limitations of this study, these findings should be considered hypothesis-generating. Prospective, adequately powered studies are required to validate these observations and define optimal dosing strategies.
